# Supplementary material for: MetaRibo-Seq measures translation in microbiomes
Source: Nat Commun. 2020 Jun 29;11:3268. doi: 10.1038/s41467-020-17081-z (PMC7324362; doi:10.1038/s41467-020-17081-z)
Supplement: Supplementary file 10 — Supplementary Data 7 [file 41467_2020_17081_MOESM10_ESM.zip › File2/Confidence_High_Taxonomy/332684_out.krona.html]

Javascript must be enabled to view this page.

members
magnitude
magnitudeUnassigned
count
unassigned
taxon
rank

332684\_out

196

196
2
superkingdom

196
976
phylum

class
200643
196

171549
order
196

family
2005525
1

genus
375288
1

1

SRS074670\_contig\_number\_241
46503
species

195
family
815

816
genus
9
195

SRS015264\_contig\_number\_20262SRS019910\_contig\_number\_contig-100\_1327.137509SRS050299\_contig\_number\_13803SRS050925\_contig\_number\_35851SRS077086\_contig\_number\_13441SRS077231\_contig\_number\_10137SRS098644\_contig\_number\_20291SRS1041129\_contig\_number\_12382SRS893295\_contig\_number\_7182

1
species
376804

SRS144753\_contig\_number\_contig-100\_2.87363

1

SRS893380\_contig\_number\_217
371601
species

1
46506

SRS018817\_contig\_number\_10017
species

species
29523

SRS147271\_contig\_number\_707
1

1
species
1638779

SRS893378\_contig\_number\_3318

2

SRS075821\_contig\_number\_937SRS893358\_contig\_number\_7849
246787
species

species

SRS011084\_contig\_number\_7664SRS011134\_contig\_number\_52756SRS011239\_contig\_number\_13067SRS013158\_contig\_number\_1181SRS013382\_contig\_number\_114SRS013687\_contig\_number\_30981SRS013951\_contig\_number\_35040SRS014412\_contig\_number\_13128SRS014534\_contig\_number\_565SRS014736\_contig\_number\_1542SRS014855\_contig\_number\_10832SRS014948\_contig\_number\_5052SRS014979\_contig\_number\_35765SRS015217\_contig\_number\_7446SRS015369\_contig\_number\_5915SRS015486\_contig\_number\_1006SRS015578\_contig\_number\_26277SRS015694\_contig\_number\_12953SRS015782\_contig\_number\_25979SRS015854\_contig\_number\_4216SRS015960\_contig\_number\_18230SRS016132\_contig\_number\_1752SRS016335\_contig\_number\_44718SRS016753\_contig\_number\_1537SRS016954\_contig\_number\_contig-100\_709.153035SRS017103\_contig\_number\_28543SRS017191\_contig\_number\_10741SRS017247\_contig\_number\_1211SRS017307\_contig\_number\_14159SRS017745\_contig\_number\_507SRS017859\_contig\_number\_894SRS018623\_contig\_number\_1794SRS019030\_contig\_number\_6353SRS019178\_contig\_number\_817SRS019267\_contig\_number\_820SRS019445\_contig\_number\_29676SRS019808\_contig\_number\_19593SRS020622\_contig\_number\_2156SRS021153\_contig\_number\_822SRS021219\_contig\_number\_1325SRS021484\_contig\_number\_12388SRS022524\_contig\_number\_14364SRS023526\_contig\_number\_14863SRS023914\_contig\_number\_12701SRS023971\_contig\_number\_1112SRS024075\_contig\_number\_239SRS024331\_contig\_number\_26712SRS024625\_contig\_number\_9356SRS045244\_contig\_number\_1361SRS046068\_contig\_number\_4751SRS047014\_contig\_number\_9496SRS047433\_contig\_number\_1884SRS048870\_contig\_number\_21778SRS049712\_contig\_number\_15283SRS049773\_contig\_number\_18381SRS049959\_contig\_number\_20325SRS050422\_contig\_number\_31870SRS050520\_contig\_number\_472SRS050752\_contig\_number\_12714SRS050941\_contig\_number\_11618SRS050974\_contig\_number\_1816SRS050998\_contig\_number\_15171SRS051031\_contig\_number\_24457SRS051610\_contig\_number\_7540SRS052697\_contig\_number\_4254SRS053335\_contig\_number\_9062SRS053356\_contig\_number\_47966SRS053398\_contig\_number\_3185SRS054059\_contig\_number\_5108SRS054905\_contig\_number\_3439SRS056259\_contig\_number\_22854SRS056537\_contig\_number\_8716SRS057480\_contig\_number\_10287SRS058723\_contig\_number\_6018SRS062427\_contig\_number\_5329SRS062701\_contig\_number\_3889SRS063040\_contig\_number\_202SRS063370\_contig\_number\_1744SRS063518\_contig\_number\_11985SRS063813\_contig\_number\_4969SRS063985\_contig\_number\_29875SRS064232\_contig\_number\_3533SRS064557\_contig\_number\_9669SRS065176\_contig\_number\_6265SRS075716\_contig\_number\_1219SRS076876\_contig\_number\_351SRS076976\_contig\_number\_12177SRS077502\_contig\_number\_4685SRS077589\_contig\_number\_2074SRS077641\_contig\_number\_13210SRS097920\_contig\_number\_27302SRS098571\_contig\_number\_13869SRS098881\_contig\_number\_68SRS104084\_contig\_number\_10971SRS1041031\_contig\_number\_20057SRS1041091\_contig\_number\_10977SRS1041095\_contig\_number\_5029SRS1041130\_contig\_number\_3202SRS1041138\_contig\_number\_11020SRS1041139\_contig\_number\_502SRS1041140\_contig\_number\_21103SRS1041141\_contig\_number\_8507SRS1041142\_contig\_number\_266SRS1041143\_contig\_number\_3895SRS1041147\_contig\_number\_1304SRS1041164\_contig\_number\_1424SRS104485\_contig\_number\_64SRS104912\_contig\_number\_14506SRS105082\_contig\_number\_8980SRS1054716\_contig\_number\_10849SRS1055022\_contig\_number\_10713SRS1055056\_contig\_number\_6461SRS1055076\_contig\_number\_973SRS1055083\_contig\_number\_11077SRS1055095\_contig\_number\_372SRS140513\_contig\_number\_15093SRS142503\_contig\_number\_32468SRS142599\_contig\_number\_22465SRS142781\_contig\_number\_160SRS142890\_contig\_number\_21702SRS142923\_contig\_number\_13781SRS142980\_contig\_number\_2085SRS143085\_contig\_number\_2134SRS143148\_contig\_number\_contig-100\_1057.154471SRS143342\_contig\_number\_151SRS143417\_contig\_number\_16329SRS143780\_contig\_number\_1232SRS143876\_contig\_number\_2852SRS144135\_contig\_number\_32892SRS144183\_contig\_number\_40875SRS144362\_contig\_number\_17549SRS144537\_contig\_number\_38785SRS146764\_contig\_number\_1641SRS146812\_contig\_number\_49712SRS146813\_contig\_number\_15702SRS147653\_contig\_number\_6891SRS147766\_contig\_number\_1150SRS147977\_contig\_number\_8427SRS148424\_contig\_number\_4758SRS148511\_contig\_number\_7961SRS148784\_contig\_number\_16147SRS148970\_contig\_number\_744SRS149244\_contig\_number\_306SRS149325\_contig\_number\_14303SRS893253\_contig\_number\_66SRS893256\_contig\_number\_1106SRS893259\_contig\_number\_5941SRS893270\_contig\_number\_18486SRS893279\_contig\_number\_10825SRS893285\_contig\_number\_450SRS893288\_contig\_number\_1632SRS893292\_contig\_number\_5786SRS893300\_contig\_number\_8499SRS893341\_contig\_number\_4254SRS893342\_contig\_number\_4509SRS893366\_contig\_number\_18329SRS971275\_contig\_number\_31767
821
157


SRS014313\_contig\_number\_3560SRS024492\_contig\_number\_14776SRS047044\_contig\_number\_14832SRS147377\_contig\_number\_8968
820
species
4

2
species
47678

SRS014287\_contig\_number\_2007SRS051882\_contig\_number\_4285

species
2292944

SRS018427\_contig\_number\_22449SRS1055038\_contig\_number\_69
2

species
671267

SRS012969\_contig\_number\_16258SRS019397\_contig\_number\_10917SRS021948\_contig\_number\_22183SRS045739\_contig\_number\_8162SRS049446\_contig\_number\_332SRS050801\_contig\_number\_2271SRS062654\_contig\_number\_12868SRS074586\_contig\_number\_4650SRS077849\_contig\_number\_14132SRS098073\_contig\_number\_4895SRS149181\_contig\_number\_31625SRS893369\_contig\_number\_7695
12

1
species

SRS014683\_contig\_number\_6652
28111

species
469590

SRS063190\_contig\_number\_11027
1
